# Substance Use Disorder-Related Disparities in Patient Experiences of Primary Care

**DOI:** 10.1089/heq.2018.0069

**Published:** 2019-05-02

**Authors:** Katherine J. Hoggatt, Susan M. Frayne, Fay S. Saechao, Elizabeth M. Yano, Donna L. Washington

**Affiliations:** ^1^ VA Health Services Research and Development (HSR&D) Center for the Study of Healthcare Innovation, Implementation & Policy, VA Greater Los Angeles Healthcare System, Sepulveda, California.; ^2^ Department of Epidemiology, UCLA Fielding School of Public Health, Los Angeles, California.; ^3^ VA HSR&D Center for Innovation to Implementation (Ci2i), VA Palo Alto Health Care System, Palo Alto, California.; ^4^ Division of Primary Care and Population Health, Stanford University School of Medicine, Stanford, California.; ^5^ Department of Health Policy and Management, UCLA Fielding School of Public Health, Los Angeles, California.; ^6^ Department of Medicine, University of California, Los Angeles Geffen School of Medicine, Los Angeles, California.

**Keywords:** health disparities, patient experience, primary care, quality measurement, substance-related disorders, veterans

## Abstract

**Purpose:** To assess disparities in primary care experiences for patients with a substance use disorder (SUD) diagnosis.

**Methods:** We assessed differences in Veterans Health Administration (VA) primary care patients' experiences using data from the 2014 outpatient VA Patient-Centered Medical Home Survey of Healthcare Experiences of Patients (SHEP; *N*=286,026). We obtained patient demographics and diagnoses from VA electronic medical record data.

**Results:** Patients with an SUD diagnosis reported worse experiences for 8 of 12 SHEP measures, including access, provider communication, and information received (*p*<0.05).

**Conclusion:** Targeted strategies may be needed to ensure patients with SUD have favorable primary care experiences.

## Introduction

Substance use disorder (SUD; abuse or dependence of alcohol or other substances^1^) is a common and costly health condition. Nearly 1 in 3 Americans meet criteria for an alcohol use disorder in their lifetime,^2^ with 1 in 10 meeting criteria for a drug use disorder,^3^ and the consequences of substance misuse and SUD cost the U.S. economy >$400 billion annually.^4^ Patients with SUD face negative health consequences related to substance misuse, which may be exacerbated when they are not receiving preventive health care. Disparities in preventive care quality have been documented for patients with SUD,^5–9^ but the forces contributing to these disparities are still being explored. Patients with SUD may face substance-use-related stigma or other barriers to engaging with primary care,^10^ which can contribute to disparities in preventive care. In addition, the broader health equity literature has cited patient–provider interactions as a major contributor to health care disparities.^11^

Initiatives such as the Veteran Health Administration (VA) Patient-Centered Medical Home (PCMH) have been promoted as a strategy to improve population health by ensuring access to high-quality preventive care and by fostering health care engagement through more personalized patient-centered care.^12^ Yet such broad efforts to improve patients' experiences may not benefit all patients equally,^13^ and documenting disparities in patients' health care experiences along nontraditional dimensions, such as SUD, is important for ensuring health care equity for all vulnerable groups. Despite considerable study on disparities in health care experiences by race/ethnicity and gender, and racial/ethnic disparities *among* patients with mental health conditions or SUD,^14^ no studies to our knowledge have assessed disparities for patients with versus without SUD across a range of patient experience measures. Our objective was to compare patient experiences of care for patients with versus without SUD.

## Methods

Our study group comprised VA patients with a primary care encounter from October 2013 to September 2014 who completed VA's PCMH Survey of Healthcare Experiences of Patients (SHEP). VA's SHEP is a mail-based survey administered monthly to a national sample of patients with recent use of VA primary care. We obtained patient experience measures covering domains from the Consumer Assessment of Healthcare Providers and Systems Clinician and Group Survey, which capture experiences relevant to PCMHs.^15^ From VA's Corporate Data Warehouse, we obtained data on patients' SUD diagnoses and demographic characteristics (age, gender, race/ethnicity, rurality of residence, and military service-connected disability rating). We limited our analysis to patients with nonmissing SHEP and SUD diagnosis data (*N*=286,026). This program evaluation study received a Determination of Non-Research from VA Greater Los Angeles Healthcare System Institutional Review Board.

### Measures

We assessed patients' health care experiences using all available SHEP measures, which included six individual measures and six composite measures. SHEP individual measures were derived from single-item survey measures: two information items (receipt of after-hours care information and reminders); three care coordination items (frequency of follow-up on test results, provider informed about specialist care, and discussion of prescriptions); and a single overall provider rating item. For the five individual SHEP items measuring receipt or frequency of a positive or timely experience for the construct measured, we dichotomized responses to “always” (or “yes”) versus any other response, which reflects how VA reports facility-level performance on these measures. For the sixth individual SHEP item, measuring overall provider rating on a 10-point scale, we dichotomized responses as 9 or 10 (the highest ratings) versus 0–8.

SHEP composites covered domains of access (getting timely appointments, care, and information), communication (how well providers communicate with patients), providers discussing medications decisions (asking about reasons for taking or not taking medications), self-management support (providers support you in taking care of your own health), comprehensiveness (providers pay attention to your mental/emotional health), and office staff interactions (helpful, courteous, and respectful office staff). Composite measures were each composed of two to six individual questions. A patient's score for each composite measure was computed as the percentage of nonmissing responses that fell in the top or most positive response category (“Always” or “Yes” or “A lot”) for the items included in that composite. Each patient's score on a SHEP composite could range from 0% to 100%, with higher scores representing more favorable patient experiences.

### Statistical analysis

Each individual measure was modeled as a binary outcome using linear binomial regression with a binary variable for presence of an SUD diagnosis as the predictor. Use of the linear binomial regression model meant that the disparity in patient experiences (i.e., difference in the proportion of patients with positive experiences, comparing patients with versus without SUD) corresponded to the estimated coefficient on the SUD diagnosis term. For the composite scores, we fit an ordinary linear regression model for each health care experience outcome with SUD diagnosis as the predictor. Disparities in patients' experiences were quantified as differences in average composite scores between patients with versus without an SUD diagnosis.

Our first set of models included the binary SUD diagnosis variable as the sole predictor. However, because demographic characteristics may be confounders of the association between SUD diagnoses and patient experiences of care,^16,17^ we also fit models that included patient age, gender, race/ethnicity, rurality of residence, and military service-connected disability rating. All models incorporated weights to account for the sampling design and for variations in the response rate by age and gender. Analyses were conducted using Stata SE, Version 15.

## Results

Among VA outpatients responding to the SHEP survey with nonmissing SUD diagnosis data (*N*=286,026), 11% had an SUD diagnosis documented in the VA electronic medical record. Patients with an SUD diagnosis were more likely to be young, male, nonwhite, and urban dwelling, and were more likely to have a 50% or greater service-connected condition, relative to patients without an SUD diagnosis (Table 1).

**Table 1.** Bivariate Association Between Substance Use Disorder Diagnosis and Demographics for Veterans Health Administration Outpatient Survey of Healthcare Experiences of Patients Survey Respondents (*N*=286,026)

	SUD diagnosis N=23,661 (%^a^)	No SUD diagnosis N=262,365 (%^a^)
Age^*^		
18–29	5.8	3.1
30–39	9.0	5.7
40–49	12.5	8.3
50–59	30.0	15.7
60–69	35.9	36.8
70–79	5.6	17.0
80+	1.3	13.4
Gender^*^		
Male	93.9	91.8
Female	6.1	8.2
Race/ethnicity^*^		
American Indian/Alaska Native	0.7	0.5
Asian	0.5	1.0
Black	26.2	15.4
Hispanic	6.9	6.1
Multirace	1.0	0.8
Native Hawaiian or other Pacific Islander	0.6	0.7
Unknown	1.9	2.0
White, non-Hispanic	62.3	73.5
Urban/rural residence^*^		
Any urban	73.9	65.8
Rural	25.2	33.1
Highly rural	0.9	1.2
Military service-connected disability rating^*^		
No service-connected disability	39.9	42.8
Service-connected 0–49%	19.9	22.5
Service-connected 50–99%	28.8	25.2
Service-connected 100%	11.4	9.5

^a^ All percentages are column percentages.

^*^ *p*<0.01 from test for association between SUD diagnosis and other characteristics.

SUD, substance use disorder.

Patients with SUD had worse health care experiences on 8 of 12 SHEP measures (*p*-values ranging from <0.001 to 0.04). These disparities were still apparent, although attenuated in magnitude, after adjusting for patient factors for 7 of the 12 measures (Table 2). Disparities exceeded five percentage points for measures capturing timely follow-up on test results, overall provider rating, and provider communication (Table 2). Patients with SUD had better experiences, relative to patients without an SUD diagnosis, for receipt of information about after-hours care and for comprehensiveness (*p*<0.01 for both). Patients with and without SUD had comparable experiences for other measures.

**Table 2.** Differences in Veterans Health Administration Patients' Favorable Primary Care Experiences, Comparing Patients with Versus Without a Substance Use Disorder Diagnosis (*N*=286,026)

	Percentage with a favorable patient experience					
	SUD diagnosis^a^ (%)	No SUD diagnosis^a^ (%)	Difference^b^ (unadjusted) (%)	p	Difference^c^ (adjusted) (%)	p
Individual measures						
Receipt of information on after-hours care	72.4	70.9	+1.5	0.01	+0.9	0.07
Receipt of reminders for tests, treatment, or appointment	81.6	80.1	+1.5	0.26	−0.1	0.80
Frequency of provider being informed about specialty care	52.4	56.4	−4.1	<0.01	−2.3	<0.01
Prescriptions discussed each visit	78.1	83.1	−4.9	<0.01	−4.2	<0.01
Timely follow-up on test results	54.6	59.9	−5.3	<0.01	−2.8	<0.01
Overall provider rating	58.6	66.5	−7.9	<0.01	−4.3	<0.01
Composite measures						
Comprehensiveness composite	69.1	61.2	+7.9	<0.01	+4.5	<0.01
Self-management support composite	58.3	57.4	+0.9	0.40	−0.1	0.82
Provider discusses medication decisions composite	58.4	60.8	−2.4	<0.01	−1.5	0.01
Office staff composite	63.5	67.2	−3.7	0.04	−0.4	0.41
Access composite	37.1	41.5	−4.4	<0.01	−2.1	<0.01
Communication composite	65.9	72.7	−6.8	<0.01	−4.8	<0.01

^a^ All percentages are column percentages.

^b^ Differences in favorable patient experiences on individual and composite measures are ordered from most positive (better experiences for patients with SUD) to most negative (better experiences for patients without SUD).

^c^ Adjusted for age, gender, race/ethnicity, rurality of residence, and military service-connected disability.

## Discussion

In a national primary care cohort, patients with SUD had worse scores on 8 of 12 measures capturing a range of health care experiences. The largest magnitude disparities (i.e., where patients with SUD had worse experiences than those without SUD) were for timely follow-up on test results, overall provider rating, and provider communication. The largest magnitude measure for which patients with SUD had *better* outcomes was the comprehensiveness composition, which assesses among other things whether patients were asked about their alcohol and drug use.

Our findings illustrate that even in an integrated health care system that promotes health care engagement, patients with SUD may still have worse patient experiences when compared with patients without SUD. Although this analysis, to our knowledge, is the first to report disparities in health care *experiences* for patients with SUD, previous studies have reported preventive health care quality shortfalls for this population.^5–9^ Further research could clarify whether patient experiences mediate some SUD disparities in care quality, thus providing a potential target for quality improvement efforts. The consistency of disparities across multiple domains also adds support to recent calls to monitor health equity not just along traditional dimensions of identity, such as race/ethnicity and gender, but also for groups who may face stigma and discrimination related to mental health conditions.^18,19^ These investigations may provide a path to improving patients' health care experiences, which may in turn improve patient engagement among vulnerable patients.

Our analysis had limitations relevant to the interpretation of these findings. Although we assessed a broad range of measures, we could not capture all important aspects of health care experiences. Understanding the dynamics of health care encounters, with the aim of improving patient experience, requires more in-depth understanding around the dimensions of care assessed with the SHEP survey measures. In addition, our measures of patient experiences were assessed cross-sectionally, and thus we could not investigate factors that may mediate patient health care experiences. Our analyses incorporated weights to account for differential response by age and gender, but we did not have data to assess and correct for potential differential response by other factors. In particular, we did not have the ability to adjust for differential response by SUD diagnosis, which may have contributed to response bias; notably, the SUD diagnosis rate among SHEP respondents (11%) was higher than among VA outpatients overall in FY2014 (9%). Finally, as with other VA studies, our findings may not necessarily generalize to veterans in non-VA care or to nonveteran patients.

## Conclusion

Efforts to improve patients' health care experiences, including VA's implementation of PCMH, have been shown to improve care overall,^20^ but may not suffice for the highest risk patients. Our findings highlight the need for health care systems to monitor patient experiences by high-risk characteristics such as SUD. Clinical innovations, including implementation of integrated addiction services,^21,22^ may be needed to ensure patients with SUD receive optimal patient-centered health care experiences.

## Abbreviations Used

OHE: Office of Health Equity
PCMH: Patient-Centered Medical Home
QUERI: Quality Enhancement Research Initiative
SHEP: Survey of Healthcare Experiences of Patients
SUD: substance use disorder
VA: Veterans Health Administration
